# Interactions between dietary phytate concentration and phytase level on phytate degradation and amino acid digestibility in broiler chickens^☆^

**DOI:** 10.1016/j.psj.2025.105468

**Published:** 2025-06-23

**Authors:** Stephanie Wolfrum, Wolfgang Siegert, Ismael Rubio-Cervantes, Tina Marie Liebhold, Dieter Feuerstein, Amélia Camarinha-Silva, Markus Rodehutscord

**Affiliations:** a Institute of Animal Science, University of Hohenheim, 70599 Stuttgart, Germany; b Department of Animal Sciences, University of Göttingen, 37077 Göttingen, Germany; c BASF SE, 67056 Ludwigshafen, Germany

**Keywords:** Phytate, Phytase, pH, Crop, Ileum

## Abstract

The objective of the present study was to investigate the effects of dietary *myo*-inositol hexakisphosphate (InsP_6_) concentration and added phytase on gastrointestinal InsP_6_ degradation, prececal digestibility of P, Ca, and amino acids (AA), and ME_N_ in broiler chickens. Dietary InsP_6_ was increased by graded substitution of corn starch with a mixture of 50 % soybean meal, 20 % rapeseed meal, 20 % sunflower meal, and 10 % rice bran (oilseed meal-rice bran level (ORL)). The experiment was arranged in a 4 × 3-factorial design with 4 ORL (leading to 1.4, 1.9, 2.4, and 3.0 g InsP_6_-P/kg) and 3 phytase levels (500, 1,500, and 3,000 FTU/kg). Male Ross 308 broilers were allocated to 84 metabolism units in groups of 10 and assigned to 1 of the 12 diets. InsP_6_ disappearance in the crop decreased with increasing ORL (45 to 24 %; *P* = 0.001). Prececal InsP_6_ disappearance and P digestibility linearly decreased with increasing ORL at 500 FTU/kg (83 to 56 % and 80 to 62 %; *P* < 0.001). Minor differences were determined for prececal InsP_6_ disappearance and P digestibility among ORL at 1,500 and 3,000 FTU/kg, but values decreased with increasing ORL (91 to 83 % and 87 to 81 %, respectively; *P* < 0.001). When prececal InsP_6_ disappearance relative to FTU was regressed against dietary InsP_6_, the relationship was non-linear at 500 FTU/kg but linear at 1,500 and 3,000 FTU/kg. Cecal InsP_6_ concentration increased with ORL and decreased with phytase (*P* < 0.001). Except for cysteine, prececal digestibility of all AA and ME_N_ decreased with increasing ORL. The data indicated that phytase supplemented at or above 1,500 FTU/kg did not limit gastrointestinal InsP_6_ degradation and AA digestibility at high InsP_6_ concentrations of the feed.

## Introduction

In plant-based diets, about two-thirds of phosphorus (P) is bound as phytic acid (*myo*-inositol (1,2,3,4,5,6) hexakisphosphate, **InsP_6_**) and its salt, phytate (Eeckhout and De Paepe, 1994). The utilization of P from InsP_6_ by broiler chickens is incomplete and highly variable (Rodehutscord et al., 2022). Therefore, hydrolyzing enzymes, such as phytases, are added to the feed to release P from InsP_6_. Upon complete dephosphorylation of InsP_6_, *myo*-inositol is released as a final product. InsP_6_ may form complexes with proteins and other nutrients in the digestive tract of broiler chickens, making them less available for digestion and absorption (Kornegay, 2001). Increased InsP_6_ degradation due to phytase supplements diminishes the formation of InsP_6_-complexes (Selle and Ravindran, 2007), thereby improving nutrient digestibility.

Protein-rich feed ingredients in poultry diets, such as soybean meal, rapeseed meal, and sunflower meal, differ in their concentration and location of InsP_6_. In soybeans, InsP_6_ is located in the protein storage vacuoles (Prattley and Stanley, 1982), while in rapeseed, InsP_6_ is located in globoids surrounded by additional protein structures inside the protein storage vacuoles (Gillespie et al., 2005). In sunflower seeds, InsP_6_ is stored in the crystalloids or globoids of the kernel (Erdmann, 1979; Miller et al., 1986). In rice grain, InsP_6_ is mainly found in the aleurone layer (Tanaka et al., 1973), and among the cereal by-products, rice bran is specifically high in InsP_6_ concentration (Eeckhout and De Paepe, 1994). Differences in storage location and concentration of InsP_6_ in feed ingredients may influence the efficiency of exogenous phytases, and hence the hydrolysis of InsP_6_ in mixed feeds (Leske and Coon, 1999). When such effects are mechanistically studied, different InsP_6_ concentrations can be achieved by adding pure InsP_6_ to avoid feed ingredient effects that could be confounding. However, working with pure InsP_6_ may not reflect the conditions of the industry when InsP_6_ originates from plant feeds. Thus, when InsP_6_ is increased in experimental diets by higher inclusion of oilseed meals or other raw materials, this must be associated with other changes to the diet, such as protein and fiber fractions, due to inherent raw material characteristics.

Broiler chickens responded differently in prececal InsP_6_ disappearance to 1,500 FTU phytase/kg of diet when either soybean meal, rapeseed meal, or sunflower meal was used as a feed ingredient (Krieg et al., 2020). In that study, calculations across all diets showed a linear relationship between the dietary InsP_6_ concentration and the prececal InsP_6_ disappearance caused by supplemented phytase: InsP_6_ disappearance at 1,500 FTU/kg increased by 0.44 nmol/FTU for each additional µmol of InsP_6_ in the feed. This indicated that the efficiency of the phytase at 1,500 FTU/kg was not influenced by the dietary InsP_6_ concentration caused by different oilseed meals in that study.

The present study aimed to verify this indication of a constant prececal InsP_6_ disappearance per unit of supplemented phytase by a systematic variation of phytase addition and InsP_6_ level using an InsP_6_-rich ingredient mixture. It was hypothesized that the dietary InsP_6_ concentration may influence the effects of supplemented phytase on InsP_6_ disappearance from the digestive tract and prececal P digestibility and P retention of broiler chickens. The first objective was to investigate the effects of dietary InsP_6_ concentration and phytase supplementation on InsP_6_ degradation and related prececal P digestibility. The second objective was to study the effects on the digestibility of amino acids (**AA**) and Ca, as well as ME_N_. The third objective was to calculate whether dietary InsP_6_ influenced the efficiency of phytase at different supplementation levels.

## Materials and methods

The experiment was conducted at the Agricultural Experimental Station “Unterer Lindenhof”, University of Hohenheim, Germany. It was approved by the Regierungspräsidium Tübingen, Germany, in accordance with German animal welfare legislation (approval No HOH 65/21_460a).

### Study design and diets

Twelve experimental diets were arranged in a 4 × 3-factorial design with 4 InsP_6_ concentrations, achieved by varying oilseed meal-rice bran levels (ORL), (ORL1, ORL2, ORL3, and ORL4 leading to 1.4, 1.9, 2.4, and 3.0 g InsP_6_-P/kg of diet) and 3 phytase levels (500, 1,500, and 3,000 FTU/kg of diet; microbial hybrid 6-phytase; Natuphos E 5000 G, BASF SE, Ludwigshafen, Germany). A wide range of InsP_6_-P concentrations was intended, with the initial 1.4 g InsP_6_-P/kg being very low but potentially relevant with the development of very low CP diets by the industry. The ORL was increased by substituting corn starch with a 50/20/20/10 blend of soybean meal, rapeseed meal, sunflower meal, and rice bran (Table 1 and Supplementary Table S1). These ingredients were chosen to represent relevant InsP_6_ sources in the industry and allowed the intended wide range of InsP_6_ concentrations of the complete feed. The exchange of ingredients caused other changes to the feed apart from its InsP_6_ concentration, for instance, in the CP and NDF concentrations (Table 2). However, by using a constant ratio of the four ingredients, such changes were consistent among all inclusion levels. All other ingredients remained unchanged. No mineral P was added to the diets and TiO_2_ was used as an indigestible marker at 5 g/kg. Essential AA concentrations at the lowest ORL were formulated at 110 % of the supply recommendations of the Gesellschaft für Ernährungsphysiologie (GfE, 1999) for 3-week-old broiler chickens, including the use of free AA sources. Non-essential free AA, including glycine, were added to the diets to avoid limitations due to insufficient non-essential AA intake (Hofmann et al., 2019). The diets were produced in the facilities of the Agricultural Experimental Station and pelleted without using steam through a die with a diameter of 3 mm and a hole length of 25 mm. Intended concentrations of InsP_6_-P, P, Ca, CP, AA, and phytase activity were confirmed by analyses (Table 2).

**Table 1:** Ingredient composition of the experimental diets (g/kg).

Oilseed meal-rice bran level^1^	1			2			3			4		
Phytase (FTU/kg)	500	1500	3000	500	1500	3000	500	1500	3000	500	1500	3000
Soybean meal	90	90	90	152	152	152	213	213	213	275	275	275
Rapeseed meal	36	36	36	61	61	61	85	85	85	110	110	110
Sunflower meal	36	36	36	61	61	61	85	85	85	110	110	110
Rice bran	18	18	18	30	30	30	43	43	43	55	55	55
Corn starch	383.2	383.2	383.2	259.2	259.2	259.2	137.2	137.2	137.2	13.2	13.2	13.2
Corn	300	300	300	300	300	300	300	300	300	300	300	300
Casein	62.5	62.5	62.5	62.5	62.5	62.5	62.5	62.5	62.5	62.5	62.5	62.5
Soybean oil	30	30	30	30	30	30	30	30	30	30	30	30
l-Lysine·HCl	1.7	1.7	1.7	1.7	1.7	1.7	1.7	1.7	1.7	1.7	1.7	1.7
dl-Methionine	3.1	3.1	3.1	3.1	3.1	3.1	3.1	3.1	3.1	3.1	3.1	3.1
l-Threonine	1.4	1.4	1.4	1.4	1.4	1.4	1.4	1.4	1.4	1.4	1.4	1.4
l-Tryptophan	0.4	0.4	0.4	0.4	0.4	0.4	0.4	0.4	0.4	0.4	0.4	0.4
l-Arginine	3.7	3.7	3.7	3.7	3.7	3.7	3.7	3.7	3.7	3.7	3.7	3.7
l-Isoleucine	0.8	0.8	0.8	0.8	0.8	0.8	0.8	0.8	0.8	0.8	0.8	0.8
l-Valine	2.5	2.5	2.5	2.5	2.5	2.5	2.5	2.5	2.5	2.5	2.5	2.5
l-Alanine	1.4	1.4	1.4	1.4	1.4	1.4	1.4	1.4	1.4	1.4	1.4	1.4
l-Asparagine·H_2_O	2.0	2.0	2.0	2.0	2.0	2.0	2.0	2.0	2.0	2.0	2.0	2.0
Glycine	4.0	4.0	4.0	4.0	4.0	4.0	4.0	4.0	4.0	4.0	4.0	4.0
Trace element premix^2^	0.5	0.5	0.5	0.5	0.5	0.5	0.5	0.5	0.5	0.5	0.5	0.5
Vitamin premix^3^	2.0	2.0	2.0	2.0	2.0	2.0	2.0	2.0	2.0	2.0	2.0	2.0
Limestone	9.2	9.2	9.2	9.2	9.2	9.2	9.2	9.2	9.2	9.2	9.2	9.2
Sodium bicarbonate	3.0	3.0	3.0	3.0	3.0	3.0	3.0	3.0	3.0	3.0	3.0	3.0
Sodium chloride	1.0	1.0	1.0	1.0	1.0	1.0	1.0	1.0	1.0	1.0	1.0	1.0
Choline chloride	2.6	2.6	2.6	2.6	2.6	2.6	2.6	2.6	2.6	2.6	2.6	2.6
Titanium dioxide	5.0	5.0	5.0	5.0	5.0	5.0	5.0	5.0	5.0	5.0	5.0	5.0

1 Oilseed meal-rice bran levels corresponding to 1.4 g InsP_6_-P/kg (ORL1), 1.9 g InsP_6_-P/kg (ORL2), 2.4 g InsP_6_-P/kg (ORL3), and 3.0 g InsP_6_-P/kg (ORL4).

2 Supplied per kg of diet: 25 mg calcium, 60 mg zinc, 25 mg iron, 80 mg manganese, 7.5 mg copper, 0.6 mg iodine, 0.2 mg selenium, 15 mg sepiolite.

3 Supplied per kg of diet: 0.6 g calcium, 10,000 IU vitamin A (retinyl acetate), 3,000 IU vitamin D_3_, 33 IU vitamin E (DL-α-tocopherol), 2.4 mg vitamin K_3_ (menadione), 100 µg biotin, 1.0 mg folic acid, 3.0 mg thiamine, 6.0 riboflavin, 6.0 mg pyridoxine, 30 µm cyanocobalamin, 50 mg niacin, 14 mg calcium d-pantothenate.

**Table 2:** Analyzed concentrations of dry matter, gross energy, nutrients, and phytase activity in experimental diets.

Oilseed meal-rice bran level^1^		1			2			3			4		
Phytase	FTU/kg	500	1500	3000	500	1500	3000	500	1500	3000	500	1500	3000
Dry matter	g/kg	909	903	907	907	906	909	911	908	909	909	912	912
Gross energy	MJ/kg	17.2	17.2	17.4	17.6	17.6	17.6	17.9	17.9	17.9	18.2	18.1	18.1
Phosphorus	g/kg	3.2	3.2	3.2	4.4	4.3	4.3	5.5	5.4	5.5	6.6	6.7	6.7
Calcium	g/kg	5.2	5.4	5.4	6.0	5.8	6.0	6.5	6.4	6.6	7.0	6.9	7.1
InsP_6_-phosphorus	g/kg	1.4	1.4	1.4	2.0	1.9	1.9	2.5	2.5	2.5	3.0	3.0	3.0
InsP_6_	µmol/g	7.7	7.7	7.5	10.7	10.5	10.3	13.3	13.3	13.6	16.3	16.1	16.1
Ins(1,2,4,5,6)P_5_	µmol/g	0.5	0.5	0.5	0.6	0.6	0.6	0.8	0.8	0.8	1.1	1.1	1.1
Ins(1,2,3,4,5)P_5_	µmol/g	0.3	0.3	0.3	0.4	0.4	0.4	0.5	0.5	0.5	0.7	0.7	0.7
Ins(1,2,3,4,6)P_5_	µmol/g	LOQ	LOQ	LOQ	LOQ	LOQ	LOQ	LOQ	LOQ	LOQ	0.3	0.3	0.3
*Myo*-inositol	µmol/g	0.5	0.5	0.5	1.0	1.0	1.0	1.0	1.0	1.0	1.5	1.5	1.5
Phytase activity	FTU/kg	330	1550	2930	370	1450	2960	310	1540	3290	360	1630	3080
Crude protein	g/kg	169	169	170	219	217	217	267	266	267	311	309	313
Alanine	g/kg	8.6	8.7	9.2	11.4	11.3	11.2	13.4	13.6	13.5	15.9	15.6	15.8
Arginine	g/kg	12.0	12.4	12.5	16.3	16.1	15.8	19.4	19.9	19.7	23.5	23.2	23.6
Aspartic acid + aspargine^2^	g/kg	15.6	15.6	16.5	21.8	21.5	21.2	26.4	26.8	26.5	31.8	31.5	31.9
Cysteine	g/kg	1.7	1.8	1.8	2.5	2.5	2.5	3.3	3.3	3.2	4.0	3.9	4.1
Glutamic acid + glutamine^2^	g/kg	31.0	31.1	32.7	42.4	41.4	40.8	50.1	50.4	50.0	59.0	58.6	59.8
Glycine	g/kg	9.5	9.7	10.2	12.6	12.4	12.5	14.7	14.8	14.8	17.4	17.0	17.3
Histidine	g/kg	4.5	4.6	4.8	6.3	6.3	6.1	7.7	7.6	7.5	9.0	8.8	9.2
Isoleucine	g/kg	7.8	8.0	8.3	10.6	10.5	10.3	12.5	12.7	12.5	14.9	14.7	14.8
Leucine	g/kg	14.2	14.4	15.1	19.0	18.8	18.5	22.3	22.6	22.3	26.2	25.9	26.2
Lysine	g/kg	11.0	10.9	11.5	14.5	14.3	14.1	16.9	17.2	16.9	19.9	19.6	19.9
Methionine	g/kg	6.6	6.6	6.9	7.7	7.6	7.6	8.5	8.4	8.5	9.5	9.3	9.5
Phenylalanine	g/kg	7.7	7.9	8.3	10.8	10.6	10.4	12.8	13.1	12.9	15.4	15.2	15.4
Proline	g/kg	12.1	12.5	12.8	16.3	15.4	15.1	17.9	18.1	17.5	20.5	20.1	20.0
Serine	g/kg	8.2	8.2	8.8	11.3	11.1	11.0	13.6	13.7	13.6	16.0	16.0	16.1
Threonine	g/kg	7.7	7.9	8.3	10.3	10.1	10.1	12.1	12.2	12.2	14.2	14.0	14.2
Tyrosine	g/kg	5.9	5.7	6.2	7.8	7.7	7.5	9.3	9.4	9.3	11.0	10.9	10.7
Valine	g/kg	11.3	11.4	11.8	14.4	14.2	14.0	16.5	16.7	16.6	19.3	19.0	19.2

LOQ: below limit of quantification (<0.3 µmol/g DM for Ins(1,2,3,4,6)P_5_).

1 Oilseed meal-rice bran levels corresponding to 1.4 g InsP_6_-P/kg (ORL1), 1.9 g InsP_6_-P/kg (ORL2), 2.4 g InsP_6_-P/kg (ORL3), and 3.0 g InsP_6_-P/kg (ORL4).

2 During acid hydrolysis, the amid residue in the side group of asparagine and glutamine is lost, and thus aspartic acid and glutamic acid are formed (Fontaine, 2003). Therefore, aspartic acid and asparagine as well as glutamic acid and glutamine were detected together. InsP: inositol phosphate; InsP_6_: phytate.

### Animals and housing

Male Ross 308 broiler hatchlings were obtained from a commercial hatchery (Brüterei Süd ZN der Bwe-Brüterei Weser Ems GmbH & Co. KG). Until 14 days of age, the broilers were raised in 4 floor pens (size: 3 × 4 m) on wood shavings and received a commercial starter diet containing (per kg) 215 g CP, 9 g Ca, 6 g P, 12.4 MJ ME, and 500 FTU of a 6-phytase (DEUKA Landkornstarter 315042025, Deutsche Tiernahrung Cremer GmbH & Co. KG, Düsseldorf). From day 14 until the end of the experiment on days 22 and 23, a total of 840 broilers were housed in 84 metabolism units (size: 1 m × 1 m) on a mesh-wired floor with 10 animals each. The metabolism units were assigned to the diets in a randomized complete block design with 7 replicates (metabolism units) per diet. Diets and water were provided for *ad libitum* consumption. The temperature in the animal house was set at 34°C and lighting was permanently switched on for the first 3 days after placement of the birds. Afterwards, the temperature decreased continuously to 24°C and the light program was 18 h light and 6 h dark per day until the end of the experiment. Birds were checked for overall appearance and health at least twice daily.

### Sampling and measurements

On days 14, 18, and 21, birds and diets were weighed on a metabolism unit basis to calculate ADFI, ADG, and gain to feed ratio (**G:F**). Samples of each diet were obtained on the same days to determine the DM concentration. Total excreta were collected in 12-h intervals from day 18 to 21 after removing feathers, skin scales, and spilled pellets from the stainless-steel trays placed underneath the metabolism units. Excreta were immediately frozen at - 20°C after each collection. Spilled feed was weighed after drying to correct ADFI on a DM basis. At the end of the experiment on days 22 and 23, birds were withdrawn from feed 2 h prior to slaughter. One hour before sampling, the feed was provided again with the intention to standardize crop fill. The birds were weighed and stunned with a gas mixture of 35 % CO_2_, 35 % N_2_, and 30 % O_2_ and euthanized by CO_2_ exposure. Crop, gizzard, duodenum, terminal ileum, and ceca were excised. Crop, gizzard, and ceca were cut open, and the content was removed using a spatula, while digesta from the duodenum was cautiously squeezed out by hand. The pH values of crop, gizzard, and duodenum contents were measured with an electrode (Mettler-Toledo GmbH, Gießen, Germany). Digesta from the ileum, defined as the terminal two-thirds of the section between Meckel’s diverticulum and 2 cm anterior to the ileo-ceco-colonic junction, was flushed out using ice-cold deionized water. All digesta samples were pooled per metabolism unit and immediately frozen at −20°C.

### Sample preparation and chemical analyses

Excreta samples were thawed at 4°C and homogenized within the metabolism unit. Excreta and contents of crop, ileum, and ceca were freeze-dried (Type Delta 2-24, Martin Christ Gefriertrocknungsanlagen GmbH, Osterode am Harz, Germany). Samples of the diets, digesta, and excreta were ground (Ultra Centrifugal Mill ZM 200, Retsch GmbH, Haan, Germany) through a 0.5-mm sieve and pulverized with a vibrating disk mill (PULVERISETTE 9, Fritsch GmbH, Idar-Oberstein, Germany). Ceca content was ground with a ball mill (Mixer Mill MM 400; Retsch GmbH, Haan; Germany). All analyses were conducted in duplicate, while the DM of the original excreta was determined in triplicate.

Diets and excreta were analyzed for DM (method no. 3.1) and N (method no. 4.1.1) following the official methods (Verband Deutscher Landwirtschaftlicher Untersuchungs- und Forschungsanstalten, 2012). Concentrations of P, Ca, and Ti in the diets, excreta, and crop and ileum digesta were determined by inductively coupled plasma-optical emission spectrometry after wet digestion according to a modified method of Boguhn et al. (2009). Inositol phosphate isomers (**InsP**) in the diets, crop, ileum, and ceca content were analyzed as described by Zeller et al. (2015) with minor modifications (Sommerfeld et al., 2018b). The Ins(1,2,6)P_3_, Ins(1,4,5)P_3_, and Ins(2,4,5)P_3_ are presented as InsP_3x_ because this assay could not distinguish these isomers. *Myo*-inositol was detected using an Agilent 5977A gas chromatography/mass spectrometry (Agilent Technologies Deutschland GmbH, Waldbronn, Germany) (Sommerfeld et al., 2018a). Phytase activity was analyzed using the method ISO 30024:2009. Diets and ileum digesta were analyzed for AA following oxidation and hydrolysis in acid conditions using an l-8900 Amino Acid Analyser (VWR, Hitachi, Tokyo, Japan) (Rodehutscord et al., 2004).

### Calculations and statistical analysis

Data on ADFI, ADG, and G:F were calculated from day 14 to 21 on a metabolism unit basis and corrected for mortality. Prececal InsP_6_ disappearance and digestibility of P, CP, AA, and Ca were determined according to the following equation:

(1) $$\text{Disappearance}/\text{digestibility}\;\left(\%\right)=\;100-100\;\times \left(\frac{\text{Ti}\;\text{diet}\;\left(g/\text{kg}\;\text{DM}\right)}{\text{Ti}\;\text{digesta}\;\left(g/\text{kg}\;\text{DM}\right)}\right)\times \left(\frac{\text{Analyte}\;\text{digesta}\;\left(g/\text{kg}\;\text{DM}\right)}{\text{Analyte}\;\text{diet}\;\left(g/\text{kg}\;\text{DM}\right)}\right)$$

Nutrient retention and efficiency of nutrient utilization were calculated using the following equations:

(2) Retention(g/d)=intake(g/d)−excretion(g/d)

and

(3) $$\text{Efficiency}\;\text{of}\;\text{utilization}\;\left(\%\right)=\;\frac{\text{retention}\;\left(g/d\right)}{\text{intake}\;\left(g/d\right)}\;\times \;100$$

ME_N_ was determined according to the following equation (GFE, 1999):

(4) $$ME_{N}\;\left(\text{kJ}/g\right)=\;\frac{\text{GE}-\text{intake}\;\left(\text{kJ}\right)\;-\;\text{GE}-\text{excretion}\;\left(\text{kJ}\right)\;-\;\left[36.5\;\left(\text{kJ}/g\right)\;\times \;N-\text{retention}\;\left(g\right)\right]}{\text{feed}\;\text{intake}\;\left(g\right)}$$

Results were statistically analyzed by a 2-way ANOVA using the MIXED procedure of the software package SAS for Windows (Version 9.4., SAS Institute, Cary, NC, USA). The metabolism unit was the statistical unit for all traits. The following model was used:

(5) $$y_{ijk}=\;a_{i}+b_{j}+{\left(ab\right)}_{ij}+c_{k}+e_{ijk}$$

where *y_ijk_* was the observation of the response trait, *a* the fixed effect of the ORL (ORL1, ORL2, ORL3, and ORL4 leading to 1.4, 1.9, 2.4, or 3.0 g InsP_6_-P/kg), *b* the fixed effect of the microbial phytase level (500, 1,500, and 3,000 FTU/kg), *(ab)_ij_* the interaction between the *i*th ORL and the *j*th phytase level, *c* the random block effect (1-7), and *e_ijk_* the residual error. All traits were initially tested with and without block effect. Block effects were finally included for all traits because the Akaike Information Criterion of this model was lower for most of the traits. Variance homogeneity and normal distribution were verified for each trait. The results are presented as least square means and SEM. Statistical significance was set at *P* ≤ 0.05. Graphs were visualized and regressions calculated by using GraphPad Prism 5.0 (GraphPad Software Inc., San Diego, CA, U.S.A).

## Results

The initial BW on day 14 averaged 470 g/bird (standard deviation 7.6). Mortality during the experiment was 1.4 % and not related to any treatment (12 dead birds in 8 treatments).

### InsP_6_ disappearance and prececal digestibility of P and Ca

**Fig. 1:**
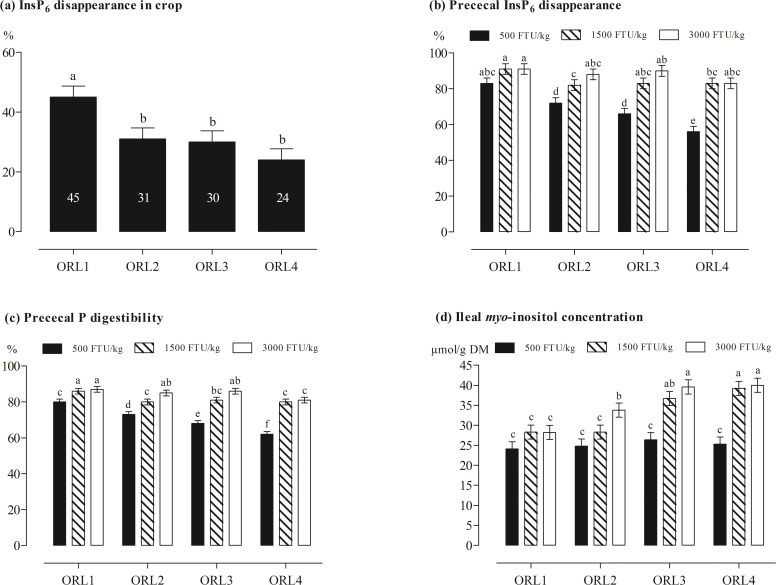
InsP_6_ disappearance in the crop (a), prececal InsP_6_ disappearance (b), prececal P digestibility (c), and ileal myo-inositol concentration (d) of broiler chickens fed diets with 4 oilseed meal-rice bran levels (ORL1, ORL2, ORL3, and ORL4 leading to 1.4, 1.9, 2.4, and 3.0 g InsP_6_-P/kg) and 3 phytase levels (500, 1,500, and 3,000 FTU/kg). Only significant (*P* ≤ 0.050) effects are shown. Columns within statistical comparison not sharing the same letter are significantly different (*P* ≤ 0.050). Further details are shown in supplementary Tables S2, S3, and S4.

In the crop content, the InsP_6_ disappearance was not significantly affected by an ORL × phytase interaction (Fig. 1(a) and Table S2). InsP_6_ disappearance in the crop significantly decreased from ORL1 to ORL2 and numerically decreased with further increased ORL (*P* = 0.001).

The ORL × phytase interaction was significant for prececal InsP_6_ disappearance and prececal P digestibility (*P* ≤ 0.007; Fig. 1(b) and (c) and Table S3). Prececal InsP_6_ disappearance and P digestibility decreased with increasing ORL at 500 FTU/kg. No difference in prececal InsP_6_ disappearance was determined within ORL when 1,500 and 3,000 FTU/kg were used, but prececal InsP_6_ disappearance decreased slightly with increasing ORL. Like the prececal InsP_6_ disappearance, prececal P digestibility was not different within ORL between 1,500 and 3,000 FTU/kg, except for a higher prececal P digestibility at ORL2 and 3,000 FTU/kg. At 1,500 FTU/kg, prececal P digestibility decreased with an increase from ORL1 to ORL2 and was unaffected with further increased ORL. No effect on prececal P digestibility was detected with an increase from ORL1 to ORL3 at 3,000 FTU/kg, but decreased at ORL4 and 3,000 FTU/kg.

The ORL × phytase interaction for prececal Ca digestibility was significant (*P* = 0.012; Table S3). Prececal Ca digestibility decreased with increasing ORL, while it was hardly affected by phytase supplementation within ORL.

**Fig. 2:**
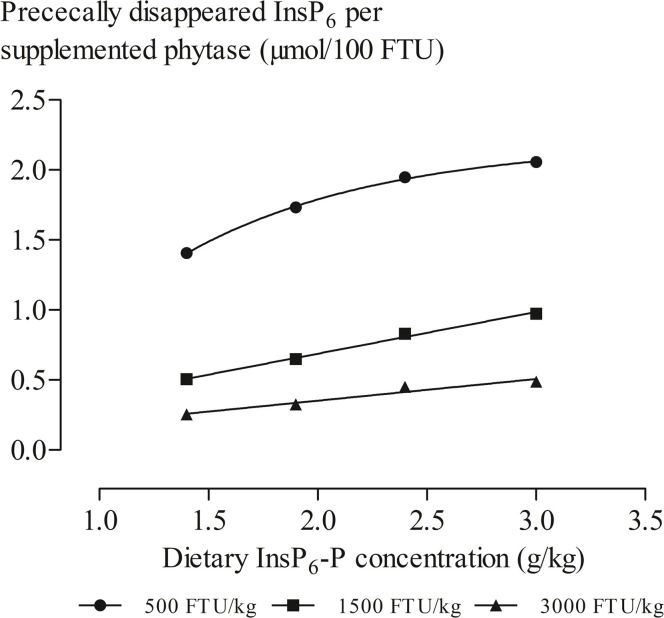
Relationship between dietary InsP_6_-P concentration (achieved by oilseed meal-rice bran level) and prececal InsP_6_ disappearance per supplemented phytase at phytase levels of 500, 1,500, 3,000 FTU/kg. Non-linear regression for 500 FTU/kg: *y* = −1.540 + 3.736 × (1-e^(−1.108x)^) (R^2^ > 0.99; Sy.*x* = 0.02). Linear regressions for 1,500 and 3,000 FTU/kg: *y* = 0.299x + 0.090 (R^2^ = 0.99; Sy.*x* = 0.02) and *y* = 0.155x + 0.042 (R^2^ = 0.95; Sy.*x* = 0.03).

A non-linear relationship was found at a phytase level of 500 FTU/kg when the prececal InsP_6_ disappearance relative to phytase was regressed against dietary InsP_6_-P concentration (Fig. 2). However, such a relationship was linear at 1,500 and 3,000 FTU/kg. Slopes of linear regressions indicated 0.299 (R^2^ = 0.99; standard deviation of the residuals (**Sy.x**) = 0.02) and 0.155 (R^2^ = 0.95; Sy.*x* = 0.03) μmol/100 FTU of prececal InsP_6_ disappearance at 1,500 and 3,000 FTU/kg, respectively.

### Inositol phosphate isomers and Myo-inositol concentrations

In the crop content, the ORL × phytase interaction was not significant for the concentrations of Ins(1,2,4,5,6)P_5_, Ins(1,2,3,4,5)P_5_, Ins(1,2,5,6)P_4_, Ins(1,2,3,4)P_4_, and InsP_3x_ (Table S2). Concentrations of Ins(1,2,4,5,6)P_5_, Ins(1,2,3,4,5)P_5_, Ins(1,2,5,6)P_4_, and Ins(1,2,3,4)P_4_ increased with increasing ORL (*P* < 0.001), while they were hardly affected by phytase supplementation (*P* ≤ 0.002). *Myo*-inositol concentration was unaffected by supplemented phytase within ORL but increased with increasing ORL (ORL × phytase: *P* = 0.035; Table S2).

In the ileal digesta, the interaction between ORL and phytase was significant for the concentrations of Ins(1,2,4,5,6)P_5_, Ins(1,2,3,4,5)P_5_, and *myo*-inositol (*P* ≤ 0.006; Table S4). At 500 FTU/kg, the Ins(1,2,4,5,6)P_5_ and Ins(1,2,3,4,5)P_5_ concentration linearly increased with increasing ORL. Concentrations of Ins(1,2,4,5,6)P_5_ and Ins(1,2,3,4,5)P_5_ were not different within ORL when 1,500 and 3,000 FTU/kg were supplemented, but both traits increased with increasing ORL. The Ins(1,2,3,4)P_4_ concentration increased with increasing ORL (*P* < 0.001). *Myo*-inositol concentration in the ileum digesta was unaffected by ORL at 500 FTU/kg (Fig. 1(d)). Minor differences were determined for the *myo*-inositol concentration between ORL levels at 1,500 and 3,000 FTU/kg, but both traits increased with increasing ORL.

In the cecal digesta, the ORL × phytase interaction was significant for the InsP_6_ concentration (*P* < 0.001; Table S5). Increasing ORL increased InsP_6_ concentration at 500 FTU/kg. The InsP_6_ concentration was not different within ORL at 1,500 and 3,000 FTU/kg, but slightly increased with increasing ORL.

### Prececal digestibility of CP and AA

**Fig. 3:**
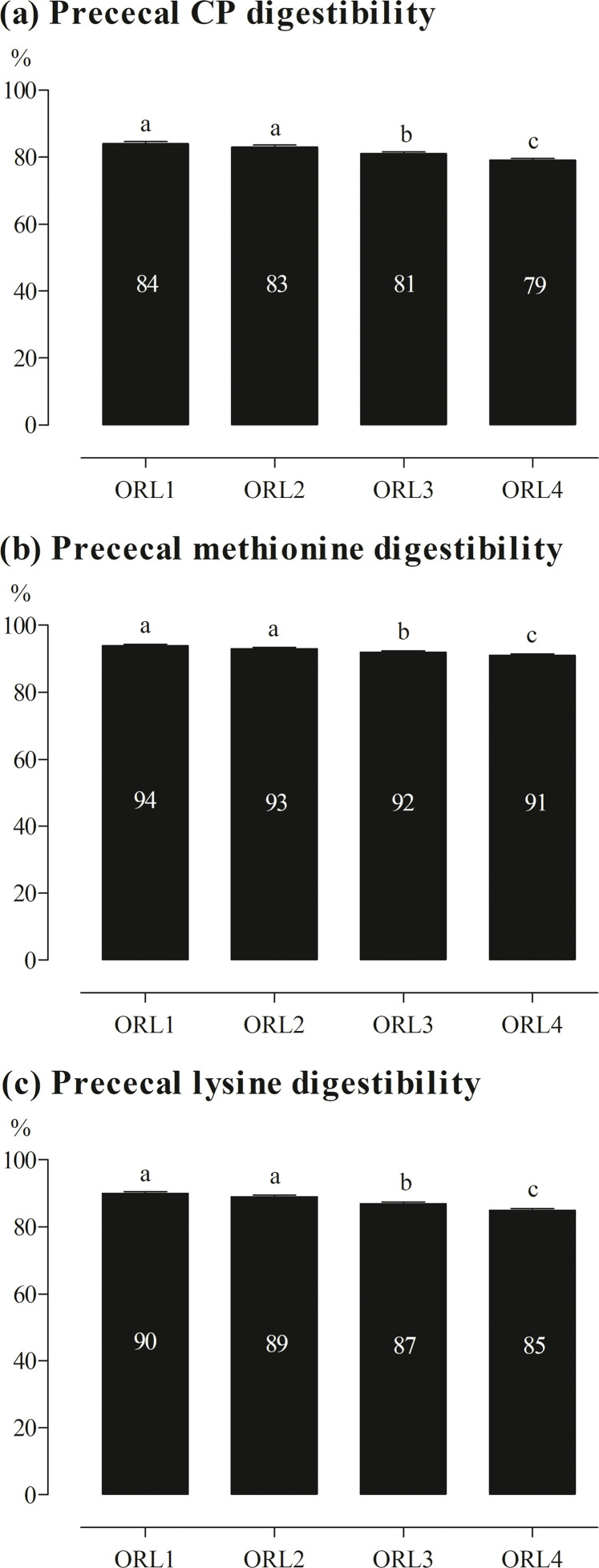
Prececal digestibility of CP (a), methionine (b), and lysine (c) of broiler chickens fed diets with 4 oilseed meal-rice bran levels (ORL1, ORL2, ORL3, and ORL4 leading to 1.4, 1.9, 2.4, and 3.0 g InsP_6_-P/kg) and 3 phytase levels (500, 1,500, and 3,000 FTU/kg). Only significant (*P* ≤ 0.050) effects are shown. Columns within statistical comparison not sharing the same letter are significantly different (*P* ≤ 0.050). Further details are shown in supplementary Table S6.

No significant interaction between ORL and phytase was determined for prececal digestibility of CP and any AA (Table S6). The values of CP, methionine, and lysine are shown in Fig. 3 as examples. Except for cysteine, prececal digestibility of CP and all AA decreased with increasing ORL by ∼4 %-points (*P* < 0.001).

### pH values of crop, gizzard, and duodenum contents

The interaction between ORL and phytase for pH values in the contents of crop, gizzard, and duodenum was not significant (Table 3). Increasing ORL increased the pH in the gizzard content (*P* < 0.001). Crop content pH significantly increased from ORL1 to ORL2 and numerically increased with further increased ORL (*P* = 0.003). The pH in the content of the gizzard was highest at a phytase supplementation of 1,500 FTU/kg and in the digesta of the duodenum at 1,500 and 3,000 FTU/kg (*P* ≤ 0.017).

**Table 3:** Effect of oilseed meal-rice bran (ORL) and phytase (Phy) levels on pH values of the crop, gizzard, and duodenum of broiler chickens.

ORL^1^	Phy	Crop	Gizzard	Duodenum
	(FTU/kg)			
*Treatments*				
1	500	5.1	3.2	5.6
1	1500	5.0	3.6	5.8
1	3000	5.2	3.3	5.9
2	500	5.2	3.8	5.7
2	1500	5.3	4.0	5.9
2	3000	5.3	3.8	5.8
3	500	5.3	3.9	5.6
3	1500	5.2	3.9	5.7
3	3000	5.3	3.8	5.8
4	500	5.4	3.7	5.7
4	1500	5.4	4.2	5.9
4	3000	5.5	4.0	5.8
Pooled SEM		0.10	0.12	0.06
*Main effects*^2^				
1		5.1^b^	3.4^d^	.
2		5.3^a^	3.9^c^	.
3		5.3^a^	3.9^b^	.
4		5.4^a^	4.0^a^	.
Pooled SEM		0.06	0.07	.
	500	.	3.7^c^	5.7^b^
	1500	.	3.9^a^	5.8^a^
	3000	.	3.7^b^	5.8^a^
	Pooled SEM	.	0.06	0.03
*ANOVA*				
ORL		0.003	<0.001	0.389
Phy		0.183	0.017	0.002
ORL × Phy		0.891	0.513	0.614

a-d Values in the same column within a statistical comparison not sharing the same subscript letter are significantly different (*P* ≤ 0.050).

1 Oilseed meal-rice bran levels corresponding to 1.4 g InsP_6_-P/kg (ORL1), 1.9 g InsP_6_-P/kg (ORL2), 2.4 g InsP_6_-P/kg (ORL3), and 3.0 g InsP_6_-P/kg (ORL4).

2 Presented if the main effect was significant (*P* ≤ 0.050) and the interaction was not significant (*P* > 0.050).

### Retention and efficiency of utilization of P, Ca, N, and ME_N_

The ORL × phytase interaction was not significant for the retention and efficiency of utilization of P, Ca, and N, as well as the ME_N_ (Table 4). The P retention increased with increasing ORL (*P* < 0.001). Retention of N increased up to ORL3 and was unaffected by further increased ORL (*P* < 0.001). The Ca retention increased from ORL1 to ORL2 and was unaffected by further increased ORL (*P* < 0.001). The efficiency of utilization of P and N and the ME_N_ decreased with increasing ORL (*P* < 0.001). The efficiency of Ca utilization increased from ORL1 to ORL2 and decreased with further increased ORL (*P* < 0.001). No differences in P and Ca retention and utilization efficiency were determined between 1,500 and 3,000 FTU/kg (*P* < 0.001).

**Table 4:** Effect of oilseed meal-rice bran (ORL) and phytase (Phy) levels on intake, retention, and efficiency of utilization of phosphorus, calcium, nitrogen, and ME_N_ of broiler chickens.

		Intake				Retention			Efficiency of utilization			
ORL^1^	Phy	P	Ca	N	GE	P	Ca	N	P	Ca	N	ME_N_
	(FTU/kg)	(g/d)			(kJ/d)	(g/d)			(%)			(kJ/g DM)
*Treatments*												
1	500	0.41	0.69	3.50	2233	0.35	0.46	2.60	86	66	74	15.7
1	1500	0.42	0.71	3.58	2282	0.38	0.51	2.64	90	72	74	15.9
1	3000	0.42	0.71	3.57	2277	0.38	0.51	2.66	91	72	75	15.8
2	500	0.57	0.79	4.67	2356	0.48	0.58	3.33	83	73	71	15.0
2	1500	0.57	0.79	4.66	2353	0.49	0.59	3.37	85	74	72	15.1
2	3000	0.58	0.80	4.69	2368	0.49	0.61	3.39	85	76	72	15.0
3	500	0.70	0.83	5.45	2287	0.50	0.56	3.64	71	68	67	14.1
3	1500	0.69	0.83	5.44	2281	0.51	0.58	3.69	73	71	68	14.0
3	3000	0.70	0.83	5.47	2293	0.51	0.59	3.66	74	71	67	14.0
4	500	0.83	0.88	6.24	2280	0.51	0.55	3.65	61	62	58	12.9
4	1500	0.84	0.88	6.25	2283	0.53	0.58	3.66	63	66	58	13.1
4	3000	0.85	0.90	6.35	2319	0.53	0.59	3.71	63	65	58	12.9
Pooled SEM		0.009	0.010	0.066	28	0.007	0.009	0.047	0.6	0.9	0.4	0.06
*Main effects*^2^												
1		0.41^d^	0.70^d^	3.55^d^	2264^b^	0.37^d^	0.49^c^	2.63^c^	89^a^	70^b^	74^a^	15.8^a^
2		0.58^c^	0.79^c^	4.67^c^	2359^a^	0.49^c^	0.59^a^	3.37^b^	84^b^	74^a^	72^b^	15.0^b^
3		0.70^b^	0.83^b^	5.45^b^	2287^b^	0.51^b^	0.58^a^^b^	3.67^a^	73^c^	70^b^	67^c^	14.1^c^
4		0.84^a^	0.89^a^	6.28^a^	2294^b^	0.52^a^	0.57^b^	3.67^a^	62^d^	64^c^	58^d^	13.0^d^
Pooled SEM		0.005	0.006	0.038	16	0.004	0.006	0.028	0.4	0.6	0.4	0.04
	500	.	.	.	.	0.46^b^	0.54^b^	.	75^b^	67^b^	.	.
	1500	.	.	.	.	0.47^a^	0.56^a^	.	78^a^	71^a^	.	.
	3000	.	.	.	.	0.48^a^	0.57^a^	.	78^a^	71^a^	.	.
	Pooled SEM	.	.	.	.	0.004	0.005	.	0.4	0.6	.	.
*ANOVA*												
ORL		<0.001	<0.001	<0.001	0.001	<0.001	<0.001	<0.001	<0.001	<0.001	<0.001	<0.001
Phy		0.504	0.465	0.495	0.448	<0.001	<0.001	0.248	<0.001	<0.001	0.120	0.071
ORL × Phy		0.971	0.960	0.970	0.947	0.958	0.494	0.988	0.225	0.370	0.110	0.630

a-d Values in the same column within a statistical comparison not sharing the same subscript letter are significantly different (*P* ≤ 0.050) GE: gross energy.

1 Oilseed meal-rice bran levels corresponding to 1.4 g InsP_6_-P/kg (ORL1), 1.9 g InsP_6_-P/kg (ORL2), 2.4 g InsP_6_-P/kg (ORL3), and 3.0 g InsP_6_-P/kg (ORL4).

2 Presented if the main effect was significant (*P* ≤ 0.050) and the interaction was not significant (*P* > 0.050).

### Performance traits

The ORL × phytase interactions were not significant for ADG, ADFI, and G:F (Table 5). The ADG increased from ORL1 to ORL2, was unaffected at ORL3, and decreased at ORL4 (*P* < 0.001). From ORL1 to ORL2, ADFI increased, but declined with further increased ORL (*P* < 0.001). The G:F ratio increased from ORL1 to ORL3 and was reduced at ORL4 (*P* < 0.001).

**Table 5:** Effect of oilseed meal-rice bran (ORL) and phytase (Phy) levels in diets on growth performance of broiler chickens from d 14 to 21 of age.

ORL^1^	Phy	ADG	ADFI	G:F
	(FTU/kg)	(g/bird)	(g/bird)	(g/g)
*Treatments*				
1	500	75	95	0.79
1	1500	78	98	0.79
1	3000	78	97	0.80
2	500	90	100	0.90
2	1500	90	100	0.90
2	3000	90	99	0.91
3	500	92	95	0.96
3	1500	91	94	0.96
3	3000	91	94	0.96
4	500	86	92	0.94
4	1500	87	91	0.95
4	3000	86	93	0.92
Pooled SEM		1.3	1.0	0.008
*Main effects*^2^				
1		77^c^	97^b^	0.79^d^
2		90^a^	100^a^	0.91^c^
3		91^a^	95^c^	0.96^a^
4		86^b^	92^d^	0.94^b^
Pooled SEM		0.8	0.6	0.005
*ANOVA*				
ORL		<0.001	<0.001	<0.001
Phy		0.780	0.813	0.748
ORL × Phy		0.638	0.348	0.262

a-d Values in the same column within a statistical comparison not sharing the same subscript letter are significantly different (*P* ≤ 0.050).

1 Oilseed meal-rice bran levels corresponding to 1.4 g InsP_6_-P/kg) (ORL1), 1.9 g InsP_6_-P/kg (ORL2), 2.4 g InsP_6_-P/kg (ORL3), and 3.0 g InsP_6_-P/kg (ORL4).

2 Presented if the main effect was significant (*P* ≤ 0.050) and the interaction was not significant (*P* > 0.050) ADFI and G:F values are standardized to 88 % DM of the feed.

## Discussion

### Effects on prececal InsP_6_ degradation

The hypothesis that the dietary InsP_6_-P concentration achieved by ORL influences the effects of the enzyme on prececal InsP_6_ disappearance and P digestibility was confirmed by the results of this study. At 500 FTU/kg, prececal InsP_6_ disappearance and P digestibility linearly decreased with increasing ORL, but the *myo*-inositol concentration in the ileum digesta was not affected. This indicated that phytase supplementation at 500 FTU/kg was the limiting factor for the hydrolysis of InsP_6_ to *myo*-inositol. Increasing ORL slightly reduced prececal InsP_6_ disappearance and P digestibility at 1,500 and 3,000 FTU/kg. A possible explanation for this reduction could be an increased formation of InsP_6_ complexes caused by rising pH in the gizzard content. Svihus (2011) reported that the quantity, retention time, and chemical characteristics of feed ingredients may lead to variable pH values in the proventriculus and gizzard of broilers. An average pH value between 3 and 4 in the gizzard was determined for the pelleted diets investigated by Svihus (2011). In the present study, the chemical characteristics of the diets were changed by substituting corn starch with soybean meal, rapeseed meal, sunflower meal, and rice bran, which possibly led to higher pH values in the gizzard, promoting the formation of InsP_6_-mineral complexes. Alterations in dietary mineral concentrations and intestinal pH could affect the solubility of InsP_6_-mineral complexes and thus the efficiency of endogenous and exogenous phytases and phosphatases (Angel et al., 2002).

Dersjant-Li et al. (2022) and Zhang et al. (2024) found no interaction effects between dietary InsP_6_-P concentration and phytase supplementation on prececal InsP_6_ disappearance, but increasing dietary InP_6_-P concentration reduced prececal InsP_6_ disappearance, while phytase supplementation increased it. The differences in dietary InsP_6_-P concentrations were achieved by the addition of rapeseed meal and rice bran (Zhang et al., 2024) and, additionally, broken rice (Dersjant-Li et al., 2022). In contrast to the present study, Zhang et al. (2024) and Dersjant-Li et al. (2022) intended to maintain similar nutrient concentrations in all diets, except for total P and InsP_6_-P, and changed the composition of diets. The present study aimed to keep the alterations in nutrient concentrations consistent across diets by using a constant ratio of the InsP_6_-rich ingredients and leaving the other ingredients, including free AA and limestone, unchanged to avoid indistinguishable effects on InsP_6_ degradation. Dersjant-Li et al. (2022) and Zhang et al. (2024) suggested that a higher inclusion level of rapeseed meal and rice bran possibly decreased prececal InsP_6_ disappearance owing to reduced overall accessibility and digestibility of InsP_6_. In a study by Leske and Coon (1999), the InsP_6_ in rice bran and rapeseed meal was less available for dephosphorylation by phytase than that in corn and soybean meal. Li et al. (2017) observed a decrease in prececal InsP_6_ disappearance with increasing dietary InsP_6_-P concentration in the presence of phytase and speculated that the inclusion of rice bran caused this decrease. In the present study, higher inclusion levels of rapeseed meal and rice bran in the diets with increasing ORL also can explain the decrease in prececal InsP_6_ disappearance and P digestibility. Since different InsP_6_ storage locations in these feed ingredients possibly resulted in reduced accessibility of InsP_6_ for degradation by supplemented phytase. Consistent with the findings of Dersjant-Li et al. (2022), Venter et al. (2024), and Zhang et al. (2024), amounts of prececal digested P increased with increasing ORL at all phytase levels in the present study (Table S3), despite decreasing percent prececal InsP_6_ disappearance and P digestibility. Dersjant-Li et al. (2022) suggested that the possibly lower InsP_6_ availability due to rapeseed meal and rice bran intake did not prevent the phytase from releasing more P. In the present study, more P was digested and *myo*-inositol in the ileum digesta increased when broilers were fed diets with increasing ORL at 1,500 and 3,000 FTU/kg, indicating that phytase did not limit dephosphorylation of InsP_6_ at increasing ORL. The increasing prececal P digestion was consistent with increasing P retention in the present study for treatments with increasing ORL.

### Effects on efficiency of supplemented phytase

When prececal InsP_6_ disappearance relative to FTU was regressed against dietary InsP_6_-P concentration, prececal InsP_6_ disappearance per unit of added phytase was the highest at the level of 500 FTU/kg. This indicated that the phytase level of 500 FTU/kg was more efficient than the phytase levels of 1,500 and 3,000 FTU/kg, while the total amount of InsP_6_ degraded by the distal ileum was highest at the highest level of phytase supplementation (Table S3). This is consistent with results of previous studies, which have shown a reduction in efficiency per unit of supplemented phytase with increasing phytase levels, as reviewed by Rodehutscord et al. (2022).

A non-linear relationship was found at a phytase level of 500 FTU/kg while linear relationships were found at phytase levels of 1,500 and 3,000 FTU/kg when the prececal InsP_6_ disappearance was regressed against dietary InsP_6_-P concentration (Fig. 2). Venter et al. (2024) observed a quadratic reduction in standardized prececal P digestibility (R^2^ = 0.92; RMSE = 0.929) with increasing dietary InsP_6_-P concentrations (1.6, 2.3, 2.8, and 3.6 g/kg), which were achieved by adding corn and corn germ, in the presence of 1,000 FTU phytase/kg. The curve calculated in that study indicated that phytase hydrolyzed the majority of InsP_6_-P at 2.3 g InsP_6_-P/kg or less, reaching a plateau at 2.9 g/kg. The concentration of InsP_6_-P in commercial diets varies depending on the raw materials used. Since 1,000 FTU/kg limited the hydrolysis of InsP_6_ at dietary InsP_6_-P above 2.3 g/kg, Venter et al. (2024) concluded that the supplemented phytase level should be adjusted to high dietary InsP_6_-P. Zhang et al. (2024) reported non-linear relationships at low and high InsP_6_-P diets (2.2 vs 3.2 g/kg; achieved by the addition of rapeseed meal and rice bran) when the digestible P was regressed against supplemented phytase levels (0, 500, 1,000, 2,000 FTU/kg, and extradoses of 3,000 and 4,000 FTU/kg for low and high InsP_6_-P diets, respectively). At 2,000 FTU/kg, the curve reached a plateau at 1.9 and 2.7 g/kg for the dietary treatments with low and high InsP_6_-P, respectively. The authors suggested that the InsP_6_-P concentration or the composition of the diet or both affected the efficiency of phytase (Zhang et al., 2024). In the present study, the non-linear response and the diminishing effect on InsP_6_ degradation indicated that at a phytase level of 500 FTU/kg the enzyme was the limiting factor for InsP_6_ hydrolysis. However, the linear responses at phytase levels above 500 FTU/kg indicated that phytase level, but not ORL, influenced the efficiency of supplemented phytase. The constant ratio of feed ingredients within an ORL and thereby constantly increasing nutrient concentrations kept the alterations consistent across diets. This may possibly explain why ORL had no effect on the efficiency of phytase.

### Effects on InsP_6_ disappearance in the crop

Increasing ORL in the present study reduced the InsP_6_ disappearance in the crop, possibly due to increasing quantities of InsP_6_ and increasing pH values, which favor the formation of InsP_6_-complexes. Protein-rich feed ingredients such as oilseed meals can increase the pH value in the digestive tract because of their high acid-binding capacities (Gilani et al., 2013). Thus, increasing levels of soybean meal, rapeseed meal, and sunflower meal likely enhanced the pH in the crop content in the present study. The crop microbiome also could have been involved. The *Lactobacillus* genus is the predominant bacterial group in the crop (Borda-Molina et al., 2016) and it is associated with reduced pH due to lactic acid production (Pertiwi and Mahendra, 2021). In a companion study that used samples from the same birds, increasing ORL reduced the abundance of *Lactobacillus* in the crop, while that of *Ligilactobacillus* and *Limosilactobacillus* was increased (Rubio-Cervantes et al., 2025). This could be a reason for alterations in the crop pH. All measured pH values in the crop were on the critical level for the formation of InsP_6_ complexes, as they are highly soluble below a pH of 4 (Grynspan and Cheryan, 1983). A lower ORL may have shifted the pH closer to the optimum of the supplemented phytase (4.5 according to the datasheet) and reduced InsP_6_-mineral complexes, leading to increased InsP_6_ disappearance.

### Effects on prececal amino acid digestibility and energy

Except for cysteine, prececal digestibility of all AA decreased with increasing ORL. This was likely caused by lower prececal digestibility of the proteins provided with the mixture of oilseed meals and rice bran compared to proteins from the other feed ingredients. Krieg et al. (2020) found lower prececal AA digestibility in rapeseed meal and sunflower meal than in soybean meal. Rapeseed meal, sunflower meal, and rice bran contain fiber fractions at a relatively high level (Senkoylu and Dale, 1999; Sharif et al., 2014), which lowers the digestibility of proteins and minerals (Jha and Mishra, 2021) and is reflected by reduced ME_N_ values with increasing ORL in the present study. Another reason for the reduction in prececal AA digestibility with increasing ORL may be an increased formation of binary and ternary protein-InsP_6_ complexes. Binary protein-InsP_6_ complexes are mainly formed in the gizzard when the pH is below 4 (Selle et al., 2000), whereas the formation of ternary InsP_6_-mineral-protein complexes is mainly dependent on the availability of Ca ions, which act as bridges between protein and InsP_6_ (Angel et al., 2002). In the present study, the pH of the gizzard content was below 4 and the increasing ORL increased the concentrations of Ca and AA in diets.

Nitrogen retention in the present study increased with increasing ORL, although the essential AA concentrations at the lowest ORL were formulated above the AA requirements. The ADG also increased from ORL1 to ORL2 and ORL3. An insufficient intake of digestible P likely limited the ADG at ORL1, because diets did not contain feed phosphates, and insufficient P intake is a well-established reason for reduced growth. Thus, enhanced N retention at ORL2 was likely caused by the increased ADG, but it did not further increase from ORL3 to ORL4 because digestible P supply was not limiting growth at these levels.

## Conclusion

Phytase supplementation at 1,500 FTU/kg or higher does not limit dephosphorylation of InsP_6_ and prececal AA digestibility with increasing ORL. The linear relationship between dietary InsP_6_-P (achieved by ORL) and prececal InsP_6_ disappearance per unit of supplemented phytase confirmed that the efficiency of supplemented phytase at 1,500 FTU/kg or higher was affected by phytase level but not ORL. Accordingly, an adjustment of phytase above this level to varying dietary InsP_6_-P concentration was not necessary under the conditions of this study.

## Funding and disclosure statement

This study was financially supported by BASF SE (Ludwigshafen, Germany). Dieter Feuerstein is an employee of BASF SE. The other authors declare no conflict of interest.

## Appendix Supplementary materials

Image, application 1

## Footnotes

Supplementary material associated with this article can be found, in the online version, at doi:10.1016/j.psj.2025.105468.
