# Supplementary material for: Interactions between dietary phytate concentration and phytase level on phytate degradation and amino acid digestibility in broiler chickens
Source: Poult Sci. 2025 Jun 23;104(9):105468. doi: 10.1016/j.psj.2025.105468 (PMC12269387; doi:10.1016/j.psj.2025.105468)
Supplement: Supplementary file 1 [file mmc1.docx]

# **Supplements to the manuscript**

# **Interactions between dietary phytate concentration and phytase level on phytate degradation and amino acid digestibility in broiler chickens**

by

Stephanie Wolfrum, Wolfgang Siegert, Ismael Rubio-Cervantes, Tina Marie Liebhold, Dieter Feuerstein, Amélia Camarinha-Silva, Markus Rodehutscord

**Table S1:** Analyzed concentrations of dry matter and nutrients in the oilseed meals and rice bran

|  |  | Soybean meal | Rapeseed meal | Sunflower meal | Rice bran |
| --- | --- | --- | --- | --- | --- |
| Dry matter | g/kg | 888 | 891 | 906 | 924 |
| Calcium | g/kg | 3.2 | 7.3 | 4.8 | 0.7 |
| Phosphorus | g/kg | 6.4 | 10.0 | 10.1 | 19.1 |
| InsP_6_-phosphorus | g/kg | 3.6 | 5.1 | 6.5 | 16.8 |
| InsP_6_ | µmol/g | 19.4 | 27.7 | 35.3 | 91.2 |
| Ins(1,2,3,4,6)P_5_ | µmol/g | LOQ | 0.8 | 0.8 | 0.8 |
| Ins(1,2,3,4,5)P_5_ | µmol/g | 0.9 | 1.6 | 2.4 | 1.8 |
| Ins(1,2,4,5,6)P_5_ | µmol/g | 1.6 | 2.5 | 2.5 | 3.3 |
| Ash | g/kg | 75 | 78 | 66 | 85 |
| Ether extract | g/kg | 21 | 36 | 19 | 162 |
| Crude protein | g/kg | 441 | 324 | 348 | 143 |
| Crude fiber | g/kg | 41 | 133 | 169 | 70 |
| NDFom | g/kg | 182 | 299 | 314 | 204 |
| ADFom | g/kg | 65 | 214 | 212 | 69 |
| Alanine | g/kg | 21.0 | 15.4 | 15.9 | 8.7 |
| Arginine | g/kg | 34.2 | 20.2 | 28.4 | 11.1 |
| Aspartic acid + asparagine^1^ | g/kg | 55.0 | 25.3 | 32.4 | 13.5 |
| Cysteine | g/kg | 6.2 | 7.9 | 5.3 | 2.9 |
| Glutamic acid + glutamine^1^ | g/kg | 86.0 | 58.2 | 68.6 | 18.6 |
| Glycine | g/kg | 20.0 | 17.5 | 20.8 | 7.7 |
| Histidine | g/kg | 13.4 | 10.1 | 9.7 | 4.1 |
| Isoleucine | g/kg | 22.1 | 14.0 | 14.3 | 5.1 |
| Leucine | g/kg | 36.7 | 24.1 | 23.2 | 10.2 |
| Lysine | g/kg | 29.4 | 19.6 | 13.0 | 7.2 |
| Methionine | g/kg | 7.1 | 7.1 | 8.4 | 3.0 |
| Phenylalanine | g/kg | 23.8 | 13.6 | 16.4 | 6.3 |
| Proline | g/kg | 23.3 | 20.8 | 14.9 | 6.3 |
| Serine | g/kg | 25.0 | 15.5 | 15.9 | 6.5 |
| Threonine | g/kg | 18.6 | 15.2 | 13.5 | 5.4 |
| Tyrosine | g/kg | 15.9 | 10.0 | 8.9 | 4.4 |
| Valine | g/kg | 22.6 | 17.9 | 17.2 | 7.6 |

LOQ: below limit of quantification (<0.3 µmol/g DM for Ins(1,2,3,4,6)P_5_)

^1^ During acid hydrolysis, the amid residue in the side group of asparagine and glutamine is lost, and thus aspartic acid and glutamic acid are formed (Fontaine, 2003)*. Therefore, aspartic acid and asparagine as well as glutamic acid and glutamine were detected together.

^*^ Fontaine, J. 2003. Amino Acid Analysis of Feeds. Pages 15-40 in Amino Acids in Animal Nutrition. J. P. F. D’Mello, ed. 2nd ed. CAB International, Wallingford, United Kingdom.

InsP: inositol phosphate; InsP_6_: phytate

**Table S2:** Effect of oilseed meal-rice bran (ORL) and phytase (Phy) levels in diets on InsP_6_ disappearance, concentrations of inositol phosphate isomers and myo-inositol in crop contents of broiler chickens

| ORL^1^ | Phy | InsP_6_ disappearance | | InsP_6_ | Ins  (1,2,4,5,6)P_5_ | Ins  (1,2,3,4,5)P_5_ | Ins  (1,2,5,6)P_4_ | Ins  (1,2,3,4)P_4_ | InsP_3x_^2^ | *Myo*-  inositol |
| --- | --- | --- | --- | --- | --- | --- | --- | --- | --- | --- |
|  | (FTU/kg) | (%) | (g/kg DM) | (µmol/g DM) | | | | | | |
| *Treatments* |  |  |  |  | | | | | | |
| 1 | 500 | 39 | 0.61 | 4.9 | 0.3 | 0.2 | 0.4 | 0.5 | 0.7 | 0.7^e^ |
| 1 | 1500 | 57 | 0.88 | 3.4 | 0.2 | LOQ | 0.2 | 0.4 | 0.8 | 0.6^e^ |
| 1 | 3000 | 40 | 0.62 | 4.8 | 0.3 | 0.2 | LOQ | 0.2 | 0.5 | 0.7^e^ |
| 2 | 500 | 27 | 0.57 | 8.3 | 0.5 | 0.4 | 0.5 | 0.6 | 0.8 | 1.0^d^ |
| 2 | 1500 | 29 | 0.61 | 8.1 | 0.5 | 0.3 | 0.5 | 0.6 | 0.9 | 1.1^d^ |
| 2 | 3000 | 36 | 0.78 | 7.2 | 0.4 | 0.3 | 0.3 | 0.4 | 0.8 | 1.1^d^ |
| 3 | 500 | 20 | 0.55 | 11.5 | 0.7 | 0.6 | 0.7 | 0.8 | 0.9 | 1.5^c^ |
| 3 | 1500 | 35 | 0.96 | 9.2 | 0.6 | 0.4 | 0.7 | 0.9 | 1.2 | 1.6^c^ |
| 3 | 3000 | 35 | 0.95 | 9.4 | 0.6 | 0.4 | 0.5 | 0.7 | 1.2 | 1.8^b^ |
| 4 | 500 | 20 | 0.64 | 14.0 | 1.0 | 0.8 | 0.8 | 0.9 | 0.9 | 2.0^a^ |
| 4 | 1500 | 25 | 0.83 | 12.6 | 0.8 | 0.7 | 1.0 | 1.1 | 1.4 | 2.1^a^ |
| 4 | 3000 | 28 | 0.93 | 12.4 | 0.8 | 0.6 | 0.7 | 0.8 | 1.1 | 2.1^a^ |
| Pooled SEM |  | 6.3 | 0.140 | 0.75 | 0.04 | 0.03 | 0.05 | 0.08 | 0.13 | 0.05 |
| *Main effects^3^* | |  |  |  |  |  |  |  |  |  |
| 1 |  | 45^a^ | . | 4.4^d^ | 0.2^d^ | - | - | 0.3^d^ | 0.7^b^ | . |
| 2 |  | 31^b^ | . | 7.9^c^ | 0.5^c^ | 0.3^c^ | 0.4^c^ | 0.5^c^ | 0.8^b^ | . |
| 3 |  | 30^b^ | . | 10.0^b^ | 0.6^b^ | 0.5^b^ | 0.7^b^ | 0.8^b^ | 1.1^a^ | . |
| 4 |  | 24^b^ | . | 13.0^a^ | 0.8^a^ | 0.7^a^ | 0.8^a^ | 0.9^a^ | 1.1^a^ | . |
| Pooled SEM |  | 3.7 | . | 0.43 | 0.03 | 0.02 | 0.03 | 0.05 | 0.08 | . |
|  | 500 | . | 0.59^b^ | 9.7^a^ | 0.6^a^ | 0.5^a^ | 0.6 | 0.7^a^ | . | . |
|  | 1500 | . | 0.82^a^ | 8.3^b^ | 0.5^b^ | - | 0.6 | 0.7^a^ | . | . |
|  | 3000 | . | 0.82^a^ | 8.5^b^ | 0.5^b^ | 0.4^b^ | - | 0.5^b^ | . | . |
|  | Pooled SEM | . | 0.070 | 0.38 | 0.02 | 0.02 | 0.03 | 0.04 | . | . |
| *ANOVA* |  |  |  |  |  |  |  |  |  |  |
| ORL |  | 0.001 | 0.421 | <0.001 | <0.001 | <0.001 | <0.001 | <0.001 | <0.001 | <0.001 |
| Phy |  | 0.061 | 0.035 | 0.025 | 0.001 | <0.001 | <0.001 | 0.002 | 0.059 | 0.002 |
| ORL×Phy |  | 0.557 | 0.635 | 0.608 | 0.160 | 0.055 | 0.090 | 0.441 | 0.500 | 0.035 |

^a-e^ Values in the same column within a statistical comparison not sharing the same subscript letter are significantly different (*P*≤0.050)

LOQ = below limit of quantification (<0.2 µmol/g DM for Ins(1,2,3,4,5)P_5_, <0.3 µmol/g DM for Ins(1,2,5,6)P_4_). Other measured inositol phosphate concentrations were below the respective detection limits in all treatments

^1^ Oilseed meal-rice bran levels corresponding to 1.4 g InsP_6_-P/kg (ORL1),1.9 g InsP_6_-P/kg (ORL2); 2.4 g InsP_6_-P/kg (ORL3), and 3.0 g InsP_6_-P/kg (ORL4)

^2^ At least one of the following isomers: Ins(1,2,6)P_3_, Ins(1,4,5)P_3_, Ins(2,4,5)P_3_

^3^ Presented if the main effect was significant (P≤0.050) and the interaction was not significant (P>0.050)

InsP: inositol phosphate; InsP_6_: phytate

**Table S3:** Effect of oilseed meal-rice bran (ORL) and phytase (Phy) levels in diets on prececal InsP_6_ disappearance and prececal digestibility of P and Ca of broiler chickens

|  |  |  | Prececal disappearance/digestibility | | | | | |
| --- | --- | --- | --- | --- | --- | --- | --- | --- |
| ORL^1^ | Phy |  | InsP_6_ | P | Ca | InsP_6_ | P | Ca |
|  | (FTU/kg) |  | (%) | | | (g/kg DM) | | |
| *Treatments* |  |  |  |  |  |  |  |  |
| 1 | 500 |  | 83^abc^ | 80^c^ | 79^a^ | 1.29^f^ | 2.81^h^ | 4.69^a^ |
| 1 | 1500 |  | 91^a^ | 86^a^ | 77^a^ | 1.41^ef^ | 3.02^h^ | 4.59^ab^ |
| 1 | 3000 |  | 91^a^ | 87^a^ | 75^a^ | 1.41^ef^ | 3.05^h^ | 4.47^abc^ |
| 2 | 500 |  | 72^d^ | 73^d^ | 64^bc^ | 1.54^de^ | 3.46^g^ | 4.17^cde^ |
| 2 | 1500 |  | 82^c^ | 80^c^ | 61^cd^ | 1.75^cd^ | 3.79^f^ | 3.97^de^ |
| 2 | 3000 |  | 88^abc^ | 85^ab^ | 66^b^ | 1.88^c^ | 4.03^ef^ | 4.33^bcd^ |
| 3 | 500 |  | 66^d^ | 68^e^ | 59^d^ | 1.79^c^ | 4.07^e^ | 4.18^cde^ |
| 3 | 1500 |  | 83^abc^ | 81^bc^ | 62^bcd^ | 2.27^b^ | 4.89^c^ | 4.43^abc^ |
| 3 | 3000 |  | 90^ab^ | 86^ab^ | 64^bc^ | 2.45^b^ | 5.16^b^ | 4.59^ab^ |
| 4 | 500 |  | 56^e^ | 62^f^ | 52^e^ | 1.85^c^ | 4.54^d^ | 4.01^de^ |
| 4 | 1500 |  | 83^bc^ | 80^c^ | 59^d^ | 2.71^a^ | 5.83^a^ | 4.57^ab^ |
| 4 | 3000 |  | 83^abc^ | 81^c^ | 59^d^ | 2.74^a^ | 5.89^a^ | 4.51^abc^ |
| Pooled SEM |  |  | 3.0 | 1.6 | 1.8 | 0.081 | 0.095 | 0.122 |
| *ANOVA* |  |  |  |  |  |  |  |  |
| ORL |  |  | <0.001 | <0.001 | <0.001 | <0.001 | <0.001 | 0.001 |
| Phy |  |  | <0.001 | <0.001 | 0.105 | <0.001 | <0.001 | 0.053 |
| ORL×Phy |  |  | 0.007 | 0.001 | 0.012 | <0.001 | <0.001 | 0.009 |

^a-h^ Values in the same column within a statistical comparison not sharing the same subscript letter are significantly different (*P*≤0.050)

^1^ Oilseed meal-rice bran levels corresponding to 1.4 g InsP_6_-P/kg (ORL1),1.9 g InsP_6_-P/kg (ORL2); 2.4 g InsP_6_-P/kg (ORL3), and 3.0 g InsP_6_-P/kg (ORL4)

InsP_6_: phytate

**Table S4:** Effect of oilseed meal-rice bran (ORL) and phytase (Phy) levels in diets on inositol phosphate isomers and myo-inositol in ileum digesta of broiler chickens

| ORL^1^ | Phy | InsP_6_ | Ins  (1,2,4,5,6)P_5_ | Ins  (1,2,3,4,5)P_5_ | Ins  (1,2,3,4,6)P_5_ | Ins  (1,2,5,6)P_4_ | Ins  (1,2,3,4)P_4_ | InsP_3x_^2^ | *Myo*-inositol |
| --- | --- | --- | --- | --- | --- | --- | --- | --- | --- |
|  | (FTU/kg) | (µmol/g DM) | | | | | | | |
| *Treatments* |  |  | | | | | | | |
| 1 | 500 | 8.5^cd^ | 0.3^ef^ | 0.6^cde^ | ND | ND | 0.3 | ND | 24.1^c^ |
| 1 | 1500 | 4.5^ef^ | 0.2^f^ | 0.2^de^ | ND | ND | ND | ND | 28.3^c^ |
| 1 | 3000 | 4.4^f^ | 0.3^f^ | 0.2^e^ | ND | ND | ND | ND | 28.2^c^ |
| 2 | 500 | 13.9^b^ | 0.7^bc^ | 1.5^b^ | LOQ | 0.3 | 1.0 | ND | 24.8^c^ |
| 2 | 1500 | 8.9^c^ | 0.5^cde^ | 0.6^cde^ | ND | 0.2 | 0.4 | ND | 28.3^c^ |
| 2 | 3000 | 6.1^cdef^ | 0.4^def^ | 0.3^de^ | ND | ND | 0.2 | ND | 33.8^b^ |
| 3 | 500 | 17.3^ab^ | 0.8^b^ | 2.0^a^ | 0.2 | 0.5 | 1.5 | 0.4 | 26.4^c^ |
| 3 | 1500 | 8.3^cde^ | 0.5^cde^ | 0.7^cd^ | LOQ | 0.2 | 0.6 | ND | 36.7^ab^ |
| 3 | 3000 | 4.8^def^ | 0.4^def^ | 0.3^de^ | ND | ND | 0.2 | ND | 39.6^a^ |
| 4 | 500 | 20.6^a^ | 1.1^a^ | 2.3^a^ | 0.3 | 0.6 | 1.7 | 0.6 | 25.3^c^ |
| 4 | 1500 | 8.6^cd^ | 0.5^cde^ | 0.8^c^ | ND | 0.3 | 0.9 | ND | 39.2^a^ |
| 4 | 3000 | 8.3^cde^ | 0.5^cd^ | 0.5^cde^ | ND | 0.3 | 0.6 | 0.3 | 40.0^a^ |
| Pooled SEM |  | 1.51 | 0.07 | 0.17 | 0.04 | 0.06 | 0.14 | 0.10 | 1.77 |
| *Main effects^3^* |  |  |  |  |  |  |  |  |  |
| 1 |  | . | . | . | . | - | - | . | . |
| 2 |  | . | . | . | . | - | 0.5^c^ | . | . |
| 3 |  | . | . | . | . | - | 0.7^b^ | . | . |
| 4 |  | . | . | . | . | 0.4 | 1.1^a^ | . | . |
| Pooled SEM |  | . | . | . | . | 0.04 | 0.09 | . | . |
|  | 500 | . | . | . | . | 0.5^a^ | 1.1 | . | . |
|  | 1500 | . | . | . | . | 0.3^b^ | - | . | . |
|  | 3000 | . | . | . | . | - | - | . | . |
|  | Pooled SEM | . | . | . | . | 0.04 | 0.08 | . | . |
| *ANOVA* |  |  |  |  |  |  |  |  |  |
| ORL |  | <0.001 | <0.001 | <0.001 | 0.377 | 0.007 | <0.001 | 0.088 | <0.001 |
| Phy |  | <0.001 | <0.001 | <0.001 | - | <0.001 | <0.001 | 0.061 | <0.001 |
| ORL×Phy |  | 0.018 | 0.003 | 0.001 | - | 0.348 | 0.407 | - | 0.006 |

^a-f^ Values in the same column within a statistical comparison not sharing the same subscript letter are significantly different (*P*≤0.050)

LOQ = below limit of quantification (<0.3 for Ins(1,2,3,4,5)P_5_); ND = below detection limit (<0.1 for Ins(1,2,3,4,6)P_5_, <0.1 for Ins(1,2,5,6)P_4_, <0.1 for Ins(1,2,3,4)P_4_, <0.6 for Ins(126, 145, 245)P_3_. Other measured inositol phosphate concentrations were below the respective detection limits in all treatments

^1^ Oilseed meal-rice bran levels corresponding to 1.4 g InsP_6_-P/kg (ORL1),1.9 g InsP_6_-P/kg (ORL2); 2.4 g InsP_6_-P/kg (ORL3), and 3.0 g InsP_6_-P/kg (ORL4)

^2^ At least one of the following isomers: Ins(1,2,6)P_3_, Ins(1,4,5)P_3_, Ins(2,4,5)P_3_

^3^ Presented if the main effect was significant (P≤0.050) and the interaction was not significant (P>0.050)

InsP: inositol phosphate; InsP_6_: phytate

**Table S5:** Effect of oilseed meal-rice bran (ORL) and phytase (Phy) levels on concentrations of inositol phosphate isomers and myo-inositol in cecal digesta of broiler chickens

| ORL^1^ | Phy | InsP_6_ | Ins(1,2,4,5,6)P_5_ | Ins(1,2,3,4,5)P_5_ | Ins(1,2,3,4)P_4_ | InsP_3x_^2^ | *Myo-*inositol |
| --- | --- | --- | --- | --- | --- | --- | --- |
|  | (FTU/kg) | (µmol/g DM) | | | | | |
| *Treatments* |  |  |  |  |  |  |  |
| 1 | 500 | 0.4^fg^ | ND | ND | ND | ND | 0.5 |
| 1 | 1500 | 0.2^g^ | ND | ND | ND | ND | 0.6 |
| 1 | 3000 | 0.2^g^ | ND | ND | ND | ND | 0.8 |
| 2 | 500 | 1.3^cd^ | ND | ND | ND | ND | 1.5 |
| 2 | 1500 | 0.6^efg^ | ND | ND | ND | ND | 0.6 |
| 2 | 3000 | 0.7^ef^ | ND | ND | ND | ND | 0.7 |
| 3 | 500 | 2.5^b^ | LOQ | 0.3 | 0.3 | 0.4 | 0.5 |
| 3 | 1500 | 1.0^de^ | ND | ND | ND | ND | 1.1 |
| 3 | 3000 | 1.0^de^ | ND | ND | ND | ND | 1.2 |
| 4 | 500 | 4.7^a^ | 0.2 | 0.5 | 0.9 | 0.8 | 0.5 |
| 4 | 1500 | 1.5^c^ | ND | ND | 0.2 | ND | 0.4 |
| 4 | 3000 | 1.0^de^ | ND | ND | ND | ND | 1.1 |
| Pooled SEM |  | 0.16 | 0.02 | 0.03 | 0.11 | 0.17 | 0.31 |
| *Main effects^3^* | |  |  |  |  |  |  |
| 1 |  | . | . | - | - | . | . |
| 2 |  | . | . | - | - | . | . |
| 3 |  | . | . | 0.3^a^ | - | . | . |
| 4 |  | . | . | 0.5^b^ | 0.5 | . | . |
| Pooled SEM |  | . | . | 0.03 | 0.08 | . | . |
|  | 500 | . | . | . | 0.6 | . | . |
|  | 1500 | . | . | . | - | . | . |
|  | 3000 | . | . | . | - | . | . |
|  | Pooled SEM | . | . | . | 0.08 | . | . |
| *ANOVA* |  |  |  |  |  |  |  |
| ORL |  | <0.001 | - | 0.001 | 0.002 | 0.104 | 0.471 |
| Phy |  | <0.001 | - | - | <0.001 | - | 0.466 |
| ORL×Phy |  | <0.001 | - | - | - | - | 0.149 |

^a-g^ Values in the same column within a statistical comparison not sharing the same subscript letter are significantly different (*P*≤0.050)

LOQ = below limit of quantification (<0.2 for Ins(1,2,4,5,6)P_5_); ND = below detection limit (<0.1 for Ins(1,2,4,5,6)P_5_, <0.1 for Ins(1,2,3,4,5)P_5_, <0.1 for Ins(1,2,3,4)P_4_, <0.3 for Ins(126, 145, 245)P_3_). Other measured inositol phosphate concentrations were below the respective detection limits in all treatments

^1^ Oilseed meal-rice bran levels corresponding to 1.4 g InsP_6_-P/kg (ORL1),1.9 g InsP_6_-P/kg (ORL2); 2.4 g InsP_6_-P/kg (ORL3), and 3.0 g InsP_6_-P/kg (ORL4)

^2^ At least one of the following isomers: Ins(1,2,6)P_3_, Ins(1,4,5)P_3_, Ins(2,4,5)P_3_

^3^ Presented if the main effect was significant (P≤0.050) and the interaction was not significant (P>0.050)

InsP: inositol phosphate; InsP_6_: phytate

**Table S6:** Effect of oilseed meal-rice bran (ORL) and phytase (Phy) levels on prececal digestibility of CP and amino acids of broiler chickens

| ORL^1^ | Phy | CP | Ala | Arg | Asx^2^ | Cys | Glx^2^ | Gly | His | Ile | Leu | Lys | Met | Phe | Pro | Ser | Thr | Tyr | Val |
| --- | --- | --- | --- | --- | --- | --- | --- | --- | --- | --- | --- | --- | --- | --- | --- | --- | --- | --- | --- |
|  | (FTU/kg) | % | | | | | | | | | | | | | | | | | |
| *Treatments* | |  |  |  |  |  |  |  |  |  |  |  |  |  |  |  |  |  |  |
| 1 | 500 | 84 | 83 | 92 | 83 | 64 | 89 | 85 | 83 | 87 | 85 | 89 | 94 | 86 | 86 | 81 | 81 | 85 | 88 |
| 1 | 1500 | 83 | 83 | 92 | 83 | 63 | 88 | 85 | 82 | 86 | 85 | 89 | 94 | 85 | 85 | 81 | 80 | 84 | 87 |
| 1 | 3000 | 84 | 84 | 92 | 84 | 63 | 89 | 85 | 82 | 87 | 85 | 90 | 94 | 86 | 85 | 81 | 81 | 85 | 88 |
| 2 | 500 | 83 | 84 | 91 | 83 | 66 | 89 | 84 | 84 | 86 | 85 | 89 | 93 | 86 | 86 | 81 | 81 | 85 | 87 |
| 2 | 1500 | 82 | 83 | 90 | 81 | 64 | 88 | 83 | 82 | 85 | 84 | 88 | 93 | 84 | 84 | 80 | 79 | 84 | 86 |
| 2 | 3000 | 83 | 84 | 91 | 83 | 66 | 89 | 84 | 83 | 86 | 85 | 89 | 93 | 86 | 85 | 81 | 80 | 85 | 87 |
| 3 | 500 | 81 | 81 | 89 | 79 | 63 | 86 | 80 | 81 | 83 | 82 | 86 | 91 | 83 | 82 | 78 | 77 | 82 | 84 |
| 3 | 1500 | 82 | 82 | 90 | 81 | 65 | 87 | 81 | 82 | 84 | 83 | 87 | 92 | 84 | 83 | 80 | 79 | 84 | 85 |
| 3 | 3000 | 81 | 82 | 89 | 80 | 64 | 87 | 81 | 81 | 84 | 83 | 87 | 92 | 84 | 83 | 79 | 78 | 83 | 85 |
| 4 | 500 | 78 | 78 | 86 | 76 | 60 | 83 | 77 | 78 | 80 | 79 | 84 | 90 | 79 | 79 | 75 | 75 | 80 | 81 |
| 4 | 1500 | 80 | 80 | 88 | 79 | 63 | 86 | 79 | 80 | 83 | 82 | 86 | 91 | 82 | 81 | 78 | 77 | 82 | 83 |
| 4 | 3000 | 80 | 81 | 88 | 79 | 65 | 86 | 79 | 81 | 83 | 82 | 86 | 91 | 83 | 81 | 79 | 78 | 83 | 84 |
| Pooled SEM | | 0.8 | 1.0 | 0.5 | 0.9 | 1.3 | 0.7 | 0.7 | 0.9 | 0.9 | 1.0 | 0.7 | 0.5 | 1.0 | 0.7 | 1.0 | 0.9 | 1.0 | 0.7 |
| *Main effects^3^* | |  |  |  |  |  |  |  |  |  |  |  |  |  |  |  |  |  |  |
| 1 |  | 84^a^ | 83^a^ | 92^a^ | 83^a^ | . | 89^a^ | 85^a^ | 83^a^ | 87^a^ | 85^a^ | 90^a^ | 94^a^ | 86^a^ | 85^a^ | 81^a^ | 81^a^ | 85^a^ | 88^a^ |
| 2 |  | 83^a^ | 83^a^ | 91^b^ | 82^b^ | . | 88^a^ | 83^b^ | 83^a^ | 86^a^ | 85^a^ | 89^a^ | 93^a^ | 85^a^ | 85^a^ | 81^a^ | 80^a^ | 84^a^ | 87^a^ |
| 3 |  | 81^b^ | 82^b^ | 89^c^ | 80^c^ | . | 87^b^ | 81^c^ | 81^b^ | 84^b^ | 83^b^ | 87^b^ | 92^b^ | 83^b^ | 83^b^ | 79^b^ | 78^b^ | 83^b^ | 85^b^ |
| 4 |  | 79^c^ | 80^c^ | 88^d^ | 78^d^ | . | 85^c^ | 79^d^ | 80^c^ | 82^c^ | 81^c^ | 85^c^ | 91^c^ | 82^c^ | 80^c^ | 77^c^ | 77^c^ | 81^c^ | 83^c^ |
| Pooled SEM | | 0.6 | 0.7 | 0.4 | 0.6 | . | 0.5 | 0.5 | 0.6 | 0.6 | 0.7 | 0.5 | 0.4 | 0.7 | 0.5 | 0.7 | 0.7 | 0.7 | 0.5 |
| *ANOVA* |  |  |  |  |  |  |  |  |  |  |  |  |  |  |  |  |  |  |  |
| ORL |  | <0.001 | <0.001 | <0.001 | <0.001 | 0.069 | <0.001 | <0.001 | <0.001 | <0.001 | <0.001 | <0.001 | <0.001 | <0.001 | <0.001 | <0.001 | <0.001 | <0.001 | <0.001 |
| Phy |  | 0.231 | 0.205 | 0.181 | 0.121 | 0.295 | 0.162 | 0.247 | 0.554 | 0.092 | 0.176 | 0.057 | 0.086 | 0.143 | 0.378 | 0.201 | 0.293 | 0.148 | 0.177 |
| ORL×Phy | | 0.272 | 0.349 | 0.075 | 0.157 | 0.361 | 0.113 | 0.229 | 0.100 | 0.169 | 0.155 | 0.150 | 0.197 | 0.129 | 0.103 | 0.172 | 0.181 | 0.198 | 0.177 |

^a-d^ Values in the same column within a statistical comparison not sharing the same subscript letter are significantly different (*P*≤0.050)

^1^ Oilseed meal-rice bran levels corresponding to 1.4 g InsP_6_-P/kg (ORL1),1.9 g InsP_6_-P/kg (ORL2); 2.4 g InsP_6_-P/kg (ORL3), and 3.0 g InsP_6_-P/kg (ORL4)

^2^ During acid hydrolysis, the amid residue in the side group of asparagine and glutamine is lost, and thus aspartic acid and glutamic acid are formed (Fontaine, 2003)*. Therefore, aspartic acid and asparagine as well as glutamic acid and glutamine were detected together

^3^ Presented if the main effect was significant (P≤0.050) and the interaction was not significant (P>0.050)

^*^ Fontaine, J. 2003. Amino Acid Analysis of Feeds. Pages 15-40 in Amino Acids in Animal Nutrition. J. P. F. D’Mello, ed. 2nd ed. CAB International, Wallingford, United Kingdom.
